# Statistical methods for handling nondetected results in food chemical monitoring data to improve food risk assessments

**DOI:** 10.1002/fsn3.3481

**Published:** 2023-07-06

**Authors:** Myungsil Hwang, Seung Chan Lee, Jae‐Hong Park, Jihee Choi, Hae‐Jeung Lee

**Affiliations:** ^1^ Department of Food and Nutrition, Institute for Aging and Clinical Nutrition Research Gachon University Seongnam‐si Korea; ^2^ Food Safety Risk Assessment Division National Institute of Food and Drug Safety Evaluation, Ministry of Food and Drug Safety Cheongju City Korea

**Keywords:** Kaplan–Meier, maximum likelihood estimation, nondetected, regression on order statistics, substitution

## Abstract

Chemical risk assessment is important for risk management, and estimates of chemical exposure must be as accurate as possible. Chemical concentrations in food below the limit of detection are known as nondetects and result in left‐censored data. During statistical analysis, the method used for handling values below the limit of detection is important. Many risk assessors employ widely used substitution methods to treat left‐censored data, as recommended by international organizations. The National Institute of Food and Drug Safety Evaluation of South Korea also recommends these methods, which are currently used for chemical exposure assessments. However, these methods have statistical limitations, and international organizations recommend more advanced alternative statistical approaches. In this study, we assessed the validity of currently used statistical methods for handling nondetects. To identify the most suitable statistical method for handling nondetection, we created virtual data and conducted simulation studies. Based on both simulation and case studies, the Maximum Likelihood Estimation (MLE) and Robust Regression on Order Statistics (ROS) methods were found to be the best options. The statistical values obtained from these methods were similar to those obtained from the commonly used 1/2 Limit of Detection (LOD) substitution method for nondetection treatment. In three case studies, we compared the various methods based on the root mean squared error. The data for all case studies were from the same source, to avoid heterogeneity. Across various sample sizes and nondetection rates, the mean and 95th percentile values for all treatment methods were similar. However, “lognormal maximum likelihood estimation” method was not suitable for estimating the mean. Risk assessors should consider statistical processing of monitoring data to reduce uncertainty. Currently used substitution methods are effective and easy to apply to large datasets with nondetection rates <80%. However, advanced statistical methods are required in some circumstances, and national guidelines are needed regarding their use in risk assessments.

## Graphical abstract

Various statistical methods are used to interpret food monitoring data, including nondetects. Statistical methods such as MLE and ROS were effective at estimating summary statisticals in datasets with NDs <80%, and results were similar to substitution method. National guidelines for the application of advanced statistical methods will help risk assessors handle nondetects.

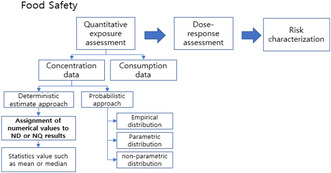

## 1 INTRODUCTION

Human lifespans have been extended due to technological advances in various areas, although exposure to various harmful substances is also increasing. With the development of advanced analysis technologies, it is possible to analyze trace amounts of hazardous substances in food, where consumer anxiety about food safety is increasing. Food is critical for maintaining health, but is a major exposure source of contaminants such as methyl mercury, inorganic arsenic, polychlorinated biphenyls, and bisphenol A (Cho et al., 2018; Farida et al., 2021; Grieb et al., 2020; Guo et al., 2019; Lars, 2003; Park et al., 2014). Typically, monitoring data distributions are not used for chronic dietary exposure assessments (FAO/WHO, 2009). This is because consumers are exposed to a range of concentration distributions over time, and their chronic exposure is associated with the mean concentration values per food (FAO/WHO, 2009). In the case of harmful substances with high nondetection rates in food, such as aflatoxins, acrylamide, and benzo(a)pyrene, proper treatment of nondetected results of analytical survey is important for chronic dietary exposure assessment. In the example of chronic dietary exposure assessment of aflatoxin B1, the detection rate of aflatoxin B1 was 4.6%, and when LOD was applied to nondetection results, the exposure was calculated as 11.8 ng/kg bw/day (NIFDS, 2021). On the other hand, the aflatoxin B1 exposure was 0.1 ng/kg bw/day when ND was zero (under bound), and this exposure level was similar to the exposure level (0.2 ng/kg bw/day) calculated only from foods with aflatoxin B1 detected in monitoring. In the 2010 evaluation, aflatoxin B1 was detected in 7.4% of food analysis results, and the exposure amount was 0.136 ng/kg bw/day (NIFDS, 2010). Exposure assessment is performed for all foods commonly consumed by the general population, and handling of nondetection values for foods with low detection rate (e.g., meat, seaweed, fish, and shellfish, etc.) may increase uncertainty in exposure assessment. In other words, the contamination level and detection rate are low, but the intake is high, so the exposure contribution can be increased.

Dietary exposure assessments combine food consumption data with data on the concentrations of hazardous chemicals in food. It is important to precisely estimate exposure amounts, from a single day to the entire lifetime, to determine the potential risk of chronic exposure (FAO/WHO, 2009). Estimates of the daily food intake of South Koreans are typically based on statistical data from the National Health and Nutrition Examination Survey (KNHANES, n.d). KNHANES is a nationwide survey of food intake using the 24‐h recall method; the data are currently used in domestic risk assessments. Data on the concentrations of potentially hazardous chemicals in domestic food are produced through large‐scale national‐level surveys and small‐scale monitoring (Kim et al., 2007, 2010, 2012; MFDS, 2021). Large‐scale monitoring of pesticides, heavy metals, and mycotoxins in frequently consumed foods is conducted regularly, but the monitoring of other hazardous chemical substances in food is insufficient.

Many hazardous substances in foods are below the limit of detection (LOD); these are referred to hereafter as nondetects (NDs) and result in a left‐censored data distribution (Farnham et al., 2002). An ND data point, i.e., an item of censored data, is not zero but nevertheless cannot be regarded as an actual concentration. If the proportion of NDs (i.e., the ND rate) is high, exposure may be under‐ or overestimated, depending on how the NDs are handled.

Typically, the mean of observed concentrations is used in equations for chronic daily exposure assessment. The risk assessor may use reported summary statistics (such as the mean, median, and interquartile range) or process raw data to calculate these values. In the latter case, the assessor should handle NDs in an appropriate way to obtain summary statistics that best represent the raw data. The NDs can affect the look of the data graphically as well as affect models, summaries, and test results. The National Institute of Food and Drug Safety Evaluation (NIFDS) recommends a substitution method proposed by international organizations for the handling of NDs; this method is currently used in chemical exposure assessments (EFSA, 2010; FAO/WHO, 2019; NIFDS, 2019). However, its compatibility with statistical techniques is limited, and more advanced alternative statistical approaches have been proposed for generating summary statistics for food chemical exposure assessments (EFSA, 2010; Helsel, 1990; USEPA, 1990).

Accurately quantifying exposure to hazardous substances in food is important for scientific food safety management. To predict general population exposure in real‐world scenarios, it is important to reduce the uncertainty caused by ND data.

The goal of this study was to identify a statistical approach that can effectively process nondetection values in analysis values of hazardous substances in food. We estimated the validity of currently used statistical methods by comparing their ability to handle NDs. Simulation studies were considered to address the bias of the NDs. After simulation for NDs based on different distributions, the simulated datasets were analyzed iteratively and the results were averaged. Finally, we compared the results of substitution method readily used in ND treatment for exposure assessment, and validated the ND treatment methods.

## 2 MATERIALS AND METHODS

### 2.1 Review of statistical approaches for handling nondetected results

This study reviewed the appropriateness of various statistical methods for handling NDs during risk assessment. We divided the approaches into the substitution methods used by international organizations such as WHO, EFSA, and more advanced alternative statistical approaches. We summarized the advantages and limitations of each method based on a review of the literature.

### 2.2 Simulation study

We randomly generated a simulated concentration dataset to compare actual and estimated values according to sample size and ND rate. We assumed three data distributions: lognormal (1, 2), gamma (1.07, 1), and Weibull (0.7, 1). Sample sizes were randomly drawn from each of the three following groups: 20–100, 100–500, and 500–1000 observations. The dataset with three different theoretical sample sizes were created by statistically 10,000 times replication. The percentage of NDs was classified as <30%, 30%–50%, or 50%–80% for each sample size class. The data were censored according to the ND rate.

The statistical methods compared in terms of their ability to handle NDs were the Kaplan–Meier (KM), regression on order statistics (ROS), and maximum likelihood estimation (MLE) methods. For MLE, the lognormal‐MLE, gamma‐MLE, and Weibull‐MLE methods were applied.

For each combination of distribution, sample size, percentage of NDs, and statistical method, summary statics, such as the mean and 95th percentile, were calculated. For each statistical method, the difference between the actual and estimated values was calculated after censoring, with true values determined based on the root mean squared error (rMSE).

For model selection using the three MLE methods, we used the Akaike information criterion (AIC) and Bayesian information criterion (BIC). The AIC is used for assessing the goodness‐of‐fit of a model using complex data (Akaike, 1972). The model having the smallest AIC value best represents the actual data distribution. The BIC uses Bayesian logic to determine the dimensions of a model for observations that have an exponential distribution and are independently and equally distributed (Schewarz, 1978). The model with the smallest BIC value best represents the data.

### 2.3 Verification through case studies

The alternative statistical methods for the simulation study were verified using actual Korean observation data of hazardous chemical substances in food. The data for the case studies were similar to those for the simulation, in terms of the number of observations and ND rate. To verify the findings of the simulation study, we conducted case studies using real mercury concentration data collected by NIFDS from 2010 to 2012 (Lim et al., 2012; NIFDS, 2010). The dataset contained mercury concentration (in μg/kg) observations from 4410 samples of 119 foods obtained via national monitoring efforts. The LOD of mercury calculated by Kaiser's equation was 0.20 μg/kg. The nine foods were selected according to ND rate most similar to the ND rate of the simulation study; pork, rice, and beef, 0%–30%; tomatoes, green onions, and pears, 30%–50%; peaches, mayonnaise, and snacks, 50%–80%. The number of observations of each food was less than 100, which was extracted from same data source. In the case studies, we applied the following statistical methods for handling NDs: the substitution methods (zero, 1/2 LOD, or LOD) recommended by international organizations; and the KM, MLE, and ROS methods, which typically had lower rMSE values in the simulation study (whichever method had the best performance). To treat the NDs of real mercury concentration data with the MLE method, three distributions such as lognormal, gamma, and Weibull were generated. The best distribution of each food was determined by AIC and BIC.

### 2.4 Statistical analysis

In this study, we used R software (version 4.0.0; R Development Core Team, 2008) for statistical processes, such as simulation data extraction, distribution estimation, and model fit comparison.

## 3 RESULTS

### 3.1 Literature review of statistical methods to handle nondetects

Handling concentration data below the LOD is important for dietary exposure assessment, especially when there is a large proportion of NDs or limited number of samples. Many researchers have attempted to clarify the statistical error implied by the presence of NDs (Currie, 1984; Finkelstein & Verma, 2001; Glass & Gray, 2001; Hawkins et al., 1991; Hornung & Reed, 1990; Lambert et al., 1991; Schmoyer et al., 1996, She, 1997). In this study, we reviewed statistical methods for analyzing NDs in chemical contamination data. There are five types of ND analysis methods: deletion, substitution, log‐probit regression, MLE, and nonparametric (EFSA, 2010). An overview of the statistical methods for NDs considered in our literature review is given in Table 1.

**TABLE 1:** Summary of the various statistical methods for generating summary statistics.

Statistical methods		Characteristics
Substitution methods		Simplest and most common approach Can lead to erroneous summary statistics when applied to datasets with a high nondetection rate Nondetects may be erroneously reported as the LOD Highly conservative Overestimates nondetects Produces inflated mean concentrations Not recommended for science‐based risk assessments Nondetects reported as zero Applicable only to chemicals that the risk assessor has determined are not likely to be present Nondetects reported as half of the LOD Used for chemicals that the risk assessor has determined may be below the LOD
Advanced statistical methods	Kaplan–Meier	Nonparametric Can estimate mean, median, and quantile values in the presence of nondetects, without distributional assumptions
	Maximum likelihood estimation	Parametric Can estimate percentiles for datasets containing values censored at more than one detection limit Requires advanced computational software
	Regression on order statistics	Parametric Recommended for analyzing left‐censored data Cannot be applied to complex datasets with multiple LOD values

Abbreviation: LOD, limit of detection.

#### 3.1.1 Deletion

With the deletion method, all NDs are eliminated from the dataset. In cases such as illegal hazardous substances in food or chemical spills, a conservative worst‐case exposure scenario is sometimes used for risk assessment by calculating the arithmetic mean. However, this is likely to result in overestimation of the frequency of occurrence and degree of contamination of foods with a chemical substance, which negatively impacts consumer awareness of risk. Thus, we do not recommend this approach for dietary risk assessment.

#### 3.1.2 Substitution

The most common substitution methods replace all NDs with a single value such as zero, the LOD, or half of the LOD (1/2 LOD) according to the percentage of NDs in the sample. This method has the advantage of being easy to implement; however, it has a weak statistical basis. Substitution is commonly used to generate descriptive statistics for international and national exposure assessments (FAO/WHO, 2009, 2019; GEMS/Food‐EURO, 1995), and the NIFDS addresses NDs with widely used substitution methods. However, the appropriateness of this approach is limited (Barringer et al., 2005; EFSA, 2010; Helsel, 2006; Hewett & Ganser, 2007; ITRC, 2013; McCarthy et al., 1997); disadvantages include a lack of consideration of the overall distribution of detected samples and overestimation of the exposure of a given food consumed in large quantities for datasets with a large proportion of NDs, when applying the “upper bound practice” (e.g., replacing NDs with the LOD). The theoretical basis and accuracy of these substitution methods are unclear. Risk assessors should consider the characteristics of the data below the LOD before including them in risk assessments.

#### 3.1.3 Advanced statistical methods

More advanced statistical methods to handle NDs include the nonparametric KM method, parametric MLE method, and parametric ROS. Our use of these methods was guided by recommendations from the World Health Organization Global Environment Monitoring System/Food‐EURO workshop (GEMS/Food‐EURO, 1995). These statistical methods to estimate concentrations below the LOD are technically superior to substitution but also require considerably more effort and expertise (Table 1).

*Nonparametric method: Kaplan–Meier*: The nonparametric KM method for the analysis of right‐censored datasets does not require any assumptions about the distribution (Helsel, 2005a, 2005b; Kaplan & Meier, 1958; Singh et al., 2006). Typically, monitoring data from food and environmental sources are left censored. The KM method was first used to analyze left‐censored concentration data in environmental studies (Schmoyer et al., 1996; She, 1997). Antweiler and Taylor (2008) reported that the KM method is the best overall technique for estimating summary statistics when the censoring rate in the datasets was less than 70%. To apply this method to left‐censored nondetected data, the detected dataset must first be converted from left‐ to right‐censored. After transforming the data, the uncensored observations are ranked from small to large, taking into account the number of censored data points between detected observations. The KM method uses only the ordinal data to obtain mean, median, and quantile values, including NDs. The advantages of this approach are that the mean can be estimated, along with the median quantile values, in the presence of NDs without the need for distributional assumptions (Tressou et al., 2004). However, the KM method tends to be insensitive to outliers, which occur frequently in food and environmental data (Antweiler & Taylor, 2008).

*Parametric method: Maximum likelihood estimation*: Parametric methods use an assumed distribution to generate summary statistics (Helsel, 1990). MLE assumes that values below the LOD and observation data follow a particular statistical distribution, and estimates distribution parameters from observed values (excluding NDs). The proportion of NDs is also considered. A value that maximizes the likelihood function is used (Fisher, 1992; Helsel, 2005a, 2005b). Data above and below the LOD are assumed to follow a lognormal, Weibull, or gamma distribution. MLE is a good analytical approach according to various bodies (EFSA, 2010; FAO/WHO, 2005, 2009).

Maximum likelihood estimation of median and interquartile range values are more precise when the observations fit the assumed distribution exactly and the sample size is large. The MLE method has the advantage that it can be applied to larger datasets (*n* = 25 or 50) even if the data do not follow a normal or lognormal distribution. However, it may produce imprecise estimates if the data do not match the observed distribution and the sample size is small (*n* = 5, 10, or 15). MLE is not suitable for estimating the mean because of the assumed lognormal distribution and inherent transformation bias when estimating the mean (Helsel, 1990).

*Parametric method: Regression on order statistics*: The ROS (also called log‐probit regression) method is recommended for analyzing left‐censored data (Hawkins et al., 1991; Singh et al., 2006). The data are sorted, and a linear relationship is assumed between the logarithm of the occurrence values and the inverse cumulative normal distribution of the observations' positions (Helsel, 2005a, 2005b). This method calculates a regression equation through log transformation and assumes that the detected data follow a lognormal distribution. It finds the values of NDs by fitting. ROS is limited because it cannot be applied to complex datasets, such as those with multiple LOD values (Hewett & Ganser, 2007). The probability of exceeding a particular ND value is calculated from the proportion of values exceeding the highest ND value in the dataset. As with the KM method, ROS indicates the positions of NDs in the data from the ratio of detected values. The probability that a value exceeds the detection limit is determined by fitting all data points to a straight line.

### 3.2 Simulation study

We conducted a simulation study to compare the statistical analysis performance of several methods applied to datasets containing various proportions of NDs. The simulated data were generated at three different censoring levels and at three different sample sizes. Table 2 lists the parameters used in the simulation study. We used three distributions commonly found in food chemical concentration data: (i) lognormal, (ii) gamma, and (iii) Weibull. We assumed that a dataset contains measurements censored at a single LOD value, to allow comparison with Korean concentration data, which are from the same source.

**TABLE 2:** Summary of parameters in the simulation study.

Data distribution	Lognormal (1, 2)
	Gamma (1.07, 1)
	Weibull (0.7, 1)
Sample size	20–100
	100–500
	500–1000
% censored	0–30
	30–50
	50–80
Statistical methods	Substitution (zero, 1/2 LOD, LOD)
	Kaplan–Meier
	Regression on order statistics
	MLE
	Lognormal‐MLE
	Gamma‐MLE
	Weibull‐MLE
Summary statistics	Mean, 95th percentile
Parameters used to identify the best fitting model when using the parametric MLE method	Akaike information criterion
	Bayesian information criterion
	Root mean square error

Abbreviations: LOD, limit of detection; MLE, maximum likelihood estimation.

We determined the best method for handling NDs based on the rMSE, which is used to deal with differences between values predicted by a model and actual observations. This difference reflects the precision of the estimation method: the smaller the rMSE, the more accurate the estimated value.

#### 3.2.1 rMSE values for the mean

After analyzing the simulated data in terms of the ND rate and sample size using the various estimation methods (KM, ROS, lognormal‐MLE, gamma‐MLE, and Weibull‐MLE), we compared the results against the original distribution. The rMSE values are listed in Table 3 as a function of the percentage of NDs in each sample. As stated above, the ND classes were <30%, 30%–50%, and 50%–80%.

**TABLE 3:** Root mean squared error for mean values calculated using the different methods with various nondetection rates and sample sizes.

Simulated distribution	Sample size	<30% nondetects					30%–50% nondetects					50%–80% nondetects				
		Kaplan–Meier	Regression on order statistics	Maximum likelihood estimation			Kaplan–Meier	Regression on order statistics	Maximum likelihood estimation			Kaplan–Meier	Regression on order statistics	Maximum likelihood estimation		
				Lognormal	Gamma	Weibull			Lognormal	Gamma	Weibull			Lognormal	Gamma	Weibull
Lognormal	20–100	2.605	2.617	2.426	2.626	1.682^a^	2.543	2.623	1.975^a^	2.667	2.000	2.293^a^	2.658	9.683	2.789	2.429
	100–500	2.596	2.606	2.426	2.615	1.679^a^	2.536	2.611	1.941^a^	2.656	2.004	2.285^a^	2.643	4.002	2.780	2.434
	500–1000	2.596	2.606	2.425	2.615	1.685^a^	2.537	2.610	1.937^a^	2.656	2.008	2.286^a^	2.640	3.066	2.780	2.437
Gamma	20–100	0.128	0.124	0.622	0.122^a^	1.465	0.196	0.135^a^	1.210	0.140	0.993	0.535	0.173^a^	3.968	0.304	0.375
	100–500	0.072	0.068	0.519	0.066^a^	1.431	0.155	0.085^a^	0.999	0.101	0.967	0.507	0.131^a^	2.554	0.294	0.356
	500–1000	0.048	0.042	0.498	0.041^a^	1.411	0.139	0.064^a^	0.950	0.089	0.955	0.494	0.116^a^	2.315	0.292	0.349
Weibull	20–100	0.220	0.217	4.887	0.216^a^	1.078	0.263	0.221	70.685	0.217^a^	0.563	0.687	0.243	41,503	0.304	0.201^a^
	100‐500	0.119	0.117	2.972	0.116^a^	0.996	0.170	0.122^a^	10.491	0.124	0.509	0.626	0.147^a^	461.625	0.257	0.189
	500–1000	0.072	0.070	2.732	0.069^a^	0.972	0.135	0.077^a^	8.653	0.084	0.495	0.602	0.108^a^	185.216	0.244	0.188

^a^ Lowest value among the estimation methods.

The rMSE for the mean was greater for the lognormal distribution than for the other distributions; this did not seem to relate to the sample size or percentage of NDs, with a similar trend seen for all types of simulated data. The Weibull‐MLE, lognormal‐MLE, and KM methods in lognormal distribution gave the lowest mean across all three ND classes.

By contrast, for the gamma and Weibull distributions, the rMSE for the mean depended on the sample size and percentage of NDs. According to the rMSE, the estimates were more precise for larger sample sizes and lower ND rates. Regarding the gamma distribution, for the 0–30% ND rate, the lowest rMSE was achieved with gamma‐MLE; for the 30%–50% and 50%–80% ND rates, the lowest rMSE was achieved with ROS (for all sample sizes). Regarding the Weibull distribution, gamma‐MLE had the lowest rMSE for the ND rate ≤ 50% in the 20–100 sample size class; for the 50%–80% ND rate, the rMSE was lowest for Weibull‐MLE. ROS showed the lowest rMSE for the 100–500 and 500–1000 sample size classes with an ND rate ≥ 30%.

#### 3.2.2 rMSE values for the 95th percentile

Table 4 lists the rMSE values for the 95th percentile. The gamma and Weibull distributions showed higher rMSE values for the 95th percentile than for the mean. This indicates lower precision when estimating extreme percentiles. Regarding the lognormal distribution, the rMSE values for the 95th percentile for the KM, ROS, and Weibull‐MLE methods were smaller than the respective values for the mean. For all distributions and sample sizes, the values for the KM and ROS methods were constant across all ND rate classes. However, the values for MLE were smaller for larger samples and increased with the ND rate. The ROS method gave the lowest rMSE values for the 95th percentile for all datasets, except when the ND ratio was <30% in the lognormal distribution.

**TABLE 4:** Root mean squared error for 95th percentile values calculated using the different methods with various nondetection rates and sample sizes.

Simulated distribution	Sample size	<30% nondetects					30%–50% nondetects					50%–80% nondetects				
		Kaplan–Meier	Regression on order statistics	Maximum likelihood estimation			Kaplan–Meier	Regression on order statistics	Maximum likelihood estimation			Kaplan–Meier	Regression on order statistics	Maximum likelihood estimation		
				Lognormal	Gamma	Weibull			Lognormal	Gamma	Weibull			Lognormal	Gamma	Weibull
Lognormal	20–100	1.275	1.092	1.559	2.211	**0.963** ^a^	1.275	**1.092** ^a^	3.130	2.930	1.299	1.275	**1.092** ^a^	4.106	3.372	1.953
	100–500	0.651	0.617	1.186	2.293	**0.539** ^a^	0.651	**0.617** ^a^	2.775	2.999	0.916	0.651	**0.617** ^a^	3.698	3.403	1.583
	500–1000	0.362	0.357	1.078	2.329	**0.343** ^a^	0.362	**0.357** ^a^	2.647	3.018	0.763	0.362	**0.357** ^a^	3.563	3.412	1.453
Gamma	20–100	0.522	**0.487** ^a^	3.055	1.222	0.532	0.522	**0.487** ^a^	4.753	2.154	1.055	0.522	**0.487** ^a^	4.711	2.770	1.613
	100–500	0.280	**0.273** ^a^	2.804	1.154	0.376	0.280	**0.273** ^a^	4.488	2.187	0.949	0.280	**0.273** ^a^	4.498	2.791	1.521
	500–1000	0.164	**0.163** ^a^	2.752	1.150	0.325	0.164	**0.163** ^a^	4.416	2.195	0.911	0.164	**0.163** ^a^	4.445	2.795	1.489
Weibull	20–100	1.222	**1.104** ^a^	9.315	4.059	1.283	1.222	**1.104** ^a^	15.422	4.453	2.697	1.222	**1.104** ^a^	15.035	4.674	4.270
	100–500	0.637	**0.614** ^a^	8.226	4.097	0.915	0.637	**0.614** ^a^	13.987	4.473	2.393	0.637	**0.614** ^a^	13.925	4.683	3.946
	500–1000	0.372	**0.368** ^a^	7.958	4.110	0.799	0.372	**0.368** ^a^	13.578	4.478	2.296	0.372	**0.368** ^a^	13.638	4.685	3.858

^a^ Bold values indicate lowest value among the estimation methods.

The most appropriate methods for handling NDs in each dataset are summarized in Table 5.

**TABLE 5:** Methods used in the simulation study for generating summary statistics.

Simulated distribution	Amount of available data	Mean			95th percentile		
		% censored			% censored		
		<30%	30%–50%	50%–80%	<30%	30%–50%	50%–80%
Lognormal	20–100	Weibull‐MLE	Lognormal‐MLE	Kaplan–Meier	Weibull‐MLE	ROS	ROS
	100–500						
	500–1000						
Gamma	20–100	Gamma‐MLE	ROS	ROS	ROS	ROS	ROS
	100–500						
	500–1000						
Weibull	20–100	Gamma‐MLE	Gamma‐MLE	Weibull‐MLE	ROS	ROS	ROS
	100–500		ROS	ROS			
	500–1000		ROS	ROS			

Abbreviations: MLE, maximum likelihood estimation; ROS, regression on order statistics.

### 3.3 Case studies with real data

#### 3.3.1 Case A: ND rate < 30%

In case A, we estimated the distribution of mercury concentrations in pork, rice, and beef samples having an ND rate < 30% (Table 6). The lowest AIC and BIC values were used as criteria for determining the best distribution for each food (Table 7). The median, mean, and 95th percentile for each food were calculated using four methods: (i) Substitution as zero, 1/2 LOD or LOD to ND (ii) Kaplan–Meier (KM), (iii) Regression on order statistics, and (iv) Maximum Likelihood Estimation (MLE) (Table 8).

**TABLE 6:** Characteristics of the datasets used in the case studies.

				Nondetection rate					
	0%–30%			30%–50%			50%–80%		
	Pork	Rice	Beef	Tomatoes	Green onions	Pears	Peaches	Mayonnaise	Snacks
*n*	54	62	58	54	54	44	38	35	78
Number of nondetects	3	7	13	17	22	20	19	23	61
% of nondetects	5.56	11.29	22.41	31.48	40.74	45.45	50	65.71	78.21

**TABLE 7:** Best distribution selection criteria for mercury concentration of each food applied to the parametric‐MLE methods.

Food^a^	Criteria	Lognormal	Gamma	Weibull
Pork	Parameter 1	1.211	0.6779	0.7756
	Parameter 2	1.4346	11.5095	6.5608
	AIC	337.64	337.74	335.72^b^
	BIC	341.76	341.86	339.84^b^
Rice	Parameter 1	1.2086	0.5664	0.6992
	Parameter 2	1.6418	15.6911	7.0177
	AIC	414.92	407.28	406.75^b^
	BIC	419.04	411.4	410.87^b^
Beef	Parameter 1	0.1554	0.4328	0.5983
	Parameter 2	1.8543	8.9925	2.6522
	AIC	300.52	292.49^b^	293.44
	BIC	304.64	296.61^b^	297.56
Tomatoes	Parameter 1	−0.525	0.247	6.187
	Parameter 2	2.711	19.930	0.771
	AIC	228.908^b^	241.135	235.995
	BIC	232.886^b^	245.113	239.973
Green onions	Parameter 1	−1.057	0.202	6.654
	Parameter 2	3.102	20.387	0.892
	AIC	209.628^b^	221.081	216.667
	BIC	213.606^b^	225.059	220.645
Pears	Parameter 1	−1.708	0.099	–
	Parameter 2	3.827	255.556	–
	AIC	168.532^b^	212.124	187.625
	BIC	172.100^b^	215.693	191.193
Peaches	Parameter 1	−2.116	0.150	0.694
	Parameter 2	2.633	13.356	2.888
	AIC	58.995^b^	94.212	77.806
	BIC	62.270^b^	97.487	81.081
Mayonnaise	Parameter 1	−3.483	0.119	1.590
	Parameter 2	3.625	7.479	2.905
	AIC	42.324^b^	56.818	52.708
	BIC	45.434^b^	59.929	55.819
Snacks	Parameter 1	−5.037	0.068	1.115
	Parameter 2	3.653	6.491	2.366
	AIC	−37.310^b^	33.995	13.344
	BIC	−32.596^b^	38.708	18.058

*Note*: Parameters 1 and 2 of the three distributions are: Lognormal: $\mu =\frac{1}{n}\sum \ln x_{i}$, $\sigma =\sqrt{\frac{1}{n-1}{\left(\sum \ln x_{i}-\mu \right)}^{2}}$; (μ,σ), Gamma: $\left(f\left(x;k,\theta \right)=\frac{1}{\Gamma \left(k\right),\theta^{k}}x^{\left(k-1\right)}e^{\left(-\frac{x}{\theta }\right)}\right):\left(k,\theta \right)$, Weibull: $\left(f\left(x;k,\lambda \right)=\frac{k}{\lambda }{\left(\frac{x}{\lambda }\right)}^{k-1}e^{{\left(-\frac{x}{\lambda }\right)}^{k}}\right):\left(k,\lambda \right)$.

Abbreviations: AIC, Akaike information criterion; BIC, Bayesian information criterion.

^a^ The source of mercury concentration data was food monitoring data from NIFDS from 2010 to 2012 (NIFDS 2010).

^b^ Lowest AIC or BIC.

**TABLE 8:** Mercury concentrations in food estimated using various statistical methods.

Case	Food (% of nondetects)	Nondetect treatment method	Median (μg/kg)	Mean (μg/kg)	95th percentile (μg/kg)
A	Pork (5.56%) (*n* = 52)	1/2 LOD substitution	4.41	7.80	29.45
		Kaplan–Meier	–	7.82	29.50
		Regression on order statistics^b^	–	7.81	21.25
		Gamma‐MLE^a^	4.45	7.80	26.87
	Rice (11.29%) (*n* = 62)	1/2 LOD substitution	3.17	8.89	39.96
		Kaplan–Meier	–	8.94	40.00
		Regression on order statistics^b^	–	8.93	39.83
		Gamma‐MLE^a^	4.48	8.89	32.65
	Beef (22.41%) (*n* = 58)	1/2 LOD substitution	1.78	3.90	18.25
		Kaplan–Meier	–	3.93	18.30
		Regression on order statistics^b^	–	3.92	16.01
		Gamma‐MLE^a^	1.54	3.89	15.73
B	Tomatoes (31.48%) (*n* = 54)	1/2 LOD substitution	0.98	4.95	29.23
		Kaplan–Meier	–	4.98	29.61
		Regression on order statistics^b^	–	4.95	29.23
		Lognormal‐MLE^a^	0.59	23.31	51.10
	Green onions (40.74%) (*n* = 54)	1/2 LOD substitution	0.81	4.15	14.37
		Kaplan–Meier	–	4.21	15.81
		Regression on order statistics^b^	–	4.18	14.37
		Lognormal‐MLE^a^	0.35	42.68	57.12
	Pears (45.45%) (*n* = 44)	1/2 LOD substitution	0.23	25.27	30.03
		Kaplan–Meier	0.22	25.32	31.95
		Regression on order statistics^b^	–	25.23	30.03
		Lognormal‐MLE^a^	0.18	274.45	98.17
C	Peaches (50%) (*n* = 38)	1/2 LOD substitution	0.16	2.04	4.35
		Kaplan–Meier^a^	NA	2.10	4.74
		Regression on order statistics^b^	–	2.02	4.35
	Mayonnaise (65.71%) (*n* = 35)	0 substitution	0.00	0.88	3.36
		1/2 LOD substitution	0.10	0.95	3.36
		LOD substitution	0.20	1.01	3.36
		Kaplan–Meier^a^	NA	1.12	3.84
		Regression on order statistics^b^	–	1.05	3.36
	Snacks (78.21%) (*n* = 78)	0 substitution	0.00	0.44	2.37
		1/2 LOD substitution	0.10	0.52	2.37
		LOD substitution	0.20	0.59	2.37
		Kaplan–Meier^a^	NA	0.61	4.82
		Regression on order statistics^b^	–	0.47	2.37

*Note*: LOD = 0.02 μg/kg.

Abbreviations: LOD, limit of detection; MLE, maximum likelihood estimation.

^a^ Model with best performance for the mean.

^b^ Model with best performance for the 95th percentile.

The mean values were similar among all methods (pork, 7.80–7.82; rice, 8.89–8.94; and beef, 3.89–3.93 μg/kg); those according to 1/2 LOD substitution and gamma‐MLE, which was the best‐performing method, were in fact identical. For case A with statistically sufficient sample size (*n* ≥ 50) and low ND rates (<30%), the mean was higher than the median. By contrast, the 95th percentile values varied depending on the method used. The substitution method values were higher than those from ROS and similar to those from KM. In summary, for case A with concentration data with an ND rate ≤ 30% and sample size >50, It is appropriate to treat NDs with 1/2 LOD substitution method to obtain the mean value for conservative risk assessment, but this is likely to overestimate the value of the 95th percentile. Risk assessors will need to consider this in the assessment of high‐exposure populations.

#### 3.3.2 Case B: ND rate of 30%–50%

The lognormal distribution gave the best estimates for tomatoes, green onions, and pears (Table 7). Therefore, lognormal‐MLE and ROS were selected to estimate the mean and 95th percentile values, respectively. The mean values obtained via the 1/2 LOD substitution, KM, and ROS methods were similar (tomatoes, 4.95–4.98; green onions, 4.14–4.21; and pears, 25.23–25.27 μg/kg), but those calculated by the lognormal‐MLE method (tomatoes, 23.31; green onions, 42.68; and pears, 274.45 μg/kg), which was the best estimation method, were up to 10 times higher (Table 8). This may be due to the uncertainty of the data increasing with the ND rate, which is inherent to lognormal distributions: when data are converted to a normal distribution through exponential transformation, the median is replaced with the geometric mean, and the mean and 95th percentile values are likely to be larger than for other distributions. Therefore, before deciding whether to use mean or 95th percentile values calculated via lognormal‐MLE for exposure assessment, it is necessary to analyze the data more closely than for other distributions. The food with higher rate of ND showed larger differences between median and mean. The 95th percentile values showed a similar pattern. In summary, for case B with a lognormal distribution and ND rate in the range of 30%–50%, the mean and 95th percentile values may be greater than those of other distributions. The treatment of NDs using the 1/2 LOD method, KM, or ROS may be useful for estimating summary statistics (mean or 95th percentile).

#### 3.3.3 Case C: ND rate of 50%–80%

The best distribution for mercury concentration levels in peaches, mayonnaise, and snacks was lognormal, according to the AIC and BIC values (Table 7). Therefore, we calculated the mean and 95th percentile values by applying the 1/2 LOD substitution, KM, and ROS methods. In the case of mayonnaise and snacks with ND ratios ≥60%, zero and LOD substitution were also applied, according to the recommendations of international organizations.

In the case of peaches, the mean mercury concentration was estimated as 2.10 μg/kg by the KM method; this was the most precise estimate (Table 8). The 95th percentile value estimated by the ROS method was 4.35 μg/kg. Both the mean and 95th percentile values estimated by the method with the highest precision in the simulation study were similar to those estimated by the 1/2 LOD substitution method. In the cases of mayonnaise and snacks, the mean values estimated by the ROS method, which had the highest precision, were similar to those estimated by LOD substitution. The 95th percentile values estimated by the ROS method were identical to those estimated by the substitution methods (zero, 1/2 LOD, and LOD). Generally, for foods with a high ND rate, if the NDs are treated as zero or subjected to LOD substitution, there is a risk of under‐ and overestimation, respectively.

## 4 DISCUSSION

This study tried to help the challenges faced by risk assessors in obtaining accurate data on chemical concentrations in food for use in dietary exposure assessments. Often, mean and maximum detected values reported in the literature are used to estimate potential risks for the general population due to the lack of raw data. However, inaccurate estimates can lead to consumer anxiety and inefficient safety management. To overcome this, risk assessors need to produce, manage, and process high‐quality data for reliable and representative national dietary exposure assessments.

Risk assessment of hazardous substances in food is important for scientific management of food safety. Trace amounts of chemicals undetectable by laboratory equipment can exist in food. When chemical concentrations are below the limit of detection (LOD) of the measuring devices, they are referred to as nondetections (NDs) (FAO/WHO, 2019). The range of ND varies depending on the precision of the analytical method used. In the past, NDs were often treated as being zero, but this approach is controversial given recent developments in analysis equipment. Analytical data on chemical substances in food is being produced more systematically than in the past, due to the harmonization of monitoring methods and the development of analysis methods. The selection of data on concentrations of chemicals in food is determined by whether an acute or chronic dietary exposure assessment is required. For chronic dietary exposure assessments, it is important to obtain accurate, consistent, and comparable food chemical concentration data (EFSA, 2019; FAO/WHO, 2019).

It is assumed that the ND result may contain the chemical in an amount below the LOD or LOQ in conservative risk assessment unless there is evidence that the food does not contain the chemical (FAO/WHO, 2019). These chemicals are mainly environmental contaminants or substances unintentionally generated during manufacturing, processing, and cooking. The detection limit for these chemicals has been set to extremely low levels in parts per trillion (ppt) or parts per billion (ppb) according to improved analytic methods. However, for genotoxic carcinogens in food, there is assumed to be no safe threshold, so very different conclusions may be drawn depending on the dataset, ND rate, and statistical methodology applied to NDs (Bevan & Harrison, 2017; Kobets et al., 2022). The number of nondetected results can impact the estimation of the mean value, as illustrated in the introduction of this study. In the 2021 assessment of aflatoxin B1 (NIFDS, 2021), the detection rate was lower than in the 2010 monitoring, but the exposure was evaluated to have increased according to the ND treatment methods (ND is substituted as LOD).

The purpose of the domestic food surveillance programs was to develop new analytical methods or re‐evaluate regulatory standards for foods (MFDS, 2021). Most testing by the Korean Food and Drug Administration is conducted for market control purposes, where values below the prescribed limit are classified as NDs. The proportion of NDs is high (>90%) for many chemical substances in several categories of food, especially those with high consumption amounts. These chemicals included pesticides and veterinary drug residues, mycotoxins (aflatoxin B1, ochratoxin, fumonisin, and zearalenone), persistent organic pollutants (dioxin, perfluorooctanoic acid, and polychlorinated biphenyls), and bisphenol A. However, Korean monitoring datasets have limitations when used for risk assessment purposes; domestic dietary exposure estimates for these hazardous substances are currently much lower than international estimates.

Domestic occurrence data, which are obtained for various purposes, should be stable across analytical methods and time. Occurrence data are also obtained for raw ingredients. To estimate the potential human health risks posed by chronic exposure to hazardous chemical substances, risk assessors require concentration data across the full range, including low concentrations. However, basic information, such as whether foods were produced domestically or imported, and the LOD, are currently insufficient. Concentrations below the LOD can be extrapolated based on the distribution of detected values. Risk assessors need to assign NDs numerical values to allow calculation of representative statistical values for the population, this process helps reduce uncertainty. When the sample size is small and the ND rate is high, more care is needed to avoid excessive uncertainty in statistical estimations. However, many assessors with a weak statistical base do not readily know how to statistically treat NDs in datasets, especially advanced statistical methods. Therefore, simple method of replacing ND with 1/2 LOD or LOD depending on the ratio of ND is often used. However, when addressing the NDs of concentration data in exposure assessment, it is necessary to consider other advanced statistical methods identified in the results of this study.

Previous studies evaluating the performance of different statistical methods to handle nondetects showed a large heterogeneity in terms of statistical methods evaluated, sample size, number of datasets employed, sample parameters, and performance statistics (Antweiler & Taylor, 2008; EFSA, 2010; Hewett & Ganser, 2007). Some studies preferred MLE methods, whereas others recommended KM method. Some studies recommended MLE when the percentage of censored data is between 50% and 80%, but this method is reported to perform badly in other studies (Antweiler & Taylor, 2008; EFSA, 2010; Helsel, 2005a, 2005b; Hewett & Ganser, 2007). Also, common options for handling nondetected food chemical concentration results are to assign a zero, LOD (or LOQ), and 1/2 LOD (or 1/2 LOQ) to ND by principles for risk assessment of chemicals in food (FAO/WHO, 2019). In this study, four of the most commonly used statistical methods were compared using simulations of real mercury concentration datasets. MLE method and ROS methods as parametric statistical methods are suggested as best methods for handling the ND in each condition of sample sizes and ND rate. The simplest method, 1/2 LOD substitution method, was found to estimate mean and 95th percentile values accurately for datasets with large sample sizes (*n* = 35–78) and ND rates (5.56%–78.21%). In addition, we found nonparametric KM method as the best‐performing method in estimation of mean for dataset which >30 sample size and <80% ND rate. The mean of the three case studies estimated by the KM method was similar to that of 1/2 LOD or LOD substitution method. This result was not consistent with previous study that the KM method showed a tendency toward underestimation of the mean for high percentages of censored data (EFSA, 2010). There are several approaches to deal with NDs for the estimation of summary statistics, and each of these methodologies has its strengths and limitations as shown in Table 1. Therefore, it needs sensitivity analysis to determine the impact of decisions about data handling on the final dietary exposure assessment. On the other hand, the higher the ND rate, the larger the difference between the median and the mean. This means that the exposure may be overestimated if the mean is used for chronic exposure assessment. One of the advantages of 1/2 LOD substitution is that it is more accessible to risk assessors than advanced statistical methods, the use of which is limited by the difficulty of calculating summary statistics (mean, standard deviation, median, 95th percentile, etc.). Advances in analytical devices capable of analyzing down to the ng/kg and ug/kg level will reduce the difference in calculated statistics by substituting 1/2 LOD or LOD for NDs. When results from all the options may be presented in the dietary exposure assessment, it needs sensitivity analysis to determine the impact of decisions about data handling on the final dietary exposure estimate.

Small sample sizes of concentration data increase uncertainty in exposure assessments, so increasing the sample size may result in more accurate exposure estimates. It may also be advantageous to focus on food items that are likely to be contaminated with the chemical of interest. In our simulation, with the same distribution and ND rate, rMSE decreased, and the precision of the estimates increased, as the number of samples increased (Tables 3 and 4). This indicates that more accurate food contamination levels can be obtained by increasing the amount of sample. It may be advantageous to isolate ingredients that are highly likely to be contaminated with a chemical substance of interest, rather than perform analysis at the whole food level. Although most risk assessors use the substitution method, its applicability is limited by the ND rate. Therefore, alternative advanced statistical methods for analyzing left‐censored data should be considered for domestic food risk assessment. Guidelines will also be needed to utilize these methods. Research is also needed on how to reduce uncertainty, to allow extrapolation of detected values to larger proportions of NDs.

## 5 CONCLUSION

Risk assessment of hazardous substances in food is important for scientific food safety management. Risk assessments, especially for low‐dose chronic exposure to hazardous substances in food, must be based on high‐quality concentration and food intake data, as well as appropriate statistical methods. Trace amounts of hazardous substances in food are below the LOD of analysis equipment, but these results are nevertheless important for exposure assessments. The performance of different statistical methods to handle nondetects showed a large heterogeneity in terms of statistical methods evaluated, sample size, number of datasets employed, sample parameters, and performance statistics. The best method to use depends on the amount of data below the LOD, the size of the dataset, and the probability distribution of the measurements. In this study, MLE method and ROS methods as parametric statistical methods suggested as best methods for handling the ND in each condition of sample sizes and ND rate. Datasets with an ND rate <80% exhibited similar or identical statistics, according to all calculation methods assessed herein. We consider 1/2 LOD substitution to be the most reliable and convenient method. In addition, the detection limit becomes lower due to the development of analytical methods, substituting 1/2 LOD for the ND value may be a more reasonable method than replacing the LOD. For datasets with an ND rate ≥ 80%, which is often seen in Korean monitoring datasets of hazardous substances in food, determining NDs using a statistical method affects the uncertainty of the evaluations. In the case of large number of NDs, distributional methods such as KM method or ROS method may be more appropriate. Before assessing the exposure to a given hazardous chemical, risk assessors need to carefully consider the optimal analysis method and sampling process for these data before performing exposure assessments. Accurate risk assessment of hazardous substances in food is fundamental for effective food safety management, so the system that can systematically produce monitoring data should be enhanced.

## AUTHOR CONTRIBUTIONS

**Myungsil Hwang:** Conceptualization (equal); data curation (equal); investigation (equal); methodology (equal); writing – original draft (lead); writing – review and editing (lead). **Seung Chan Lee:** Formal analysis (equal); methodology (equal); software (equal); validation (equal); writing – original draft (equal); writing – review and editing (supporting). **Jae‐Hong Park:** Methodology (equal); validation (supporting); writing – review and editing (supporting). **Ji‐Hee Choi:** Investigation (supporting); methodology (supporting); writing – review and editing (supporting). **Hae‐Jeung Lee:** Conceptualization (lead); data curation (equal); funding acquisition (lead); project administration (lead); writing – original draft (equal); writing – review and editing (equal).

## Data Availability

The datasets used and/or analyzed during the current study available from the corresponding author on reasonable request.
